# Possible Value of Faecal Immunochemical Test (FIT) When Added in Symptomatic Patients Referred for Colonoscopy: A Systematic Review

**DOI:** 10.3390/cancers15072011

**Published:** 2023-03-28

**Authors:** Henrike Jacoba Brands, Brigit Van Dijk, Richard M. Brohet, Henderik L. van Westreenen, Jan Willem B. de Groot, Leon M. G. Moons, Wouter H. de Vos tot Nederveen Cappel

**Affiliations:** 1 Gastroenterology and Hepatology, Isala Hospital, 8025 AB Zwolle, The Netherlands; 2 Department of Epidemiology and Statistics, Isala Hospital, 8025 AB Zwolle, The Netherlands; 3 Abdominal Surgery, Isala Hospital, 8025 AB Zwolle, The Netherlands; 4 Isala Oncology Center, Isala Hospital, 8025 AB Zwolle, The Netherlands; 5 Gastroenterology and Hepatology, Universitair Medisch Centrum Utrecht, 3584 CX Utrecht, The Netherlands

**Keywords:** colorectal cancer, faecal immunochemical test (FIT), symptoms

## Abstract

**Simple Summary:**

Literature has shown that the correlation between intestinal complaints and the gain of colonoscopy regarding colorectal cancer (CRC) is poor. Adding a faecal immunochemical test (FIT) might improve triage of colonoscopy. The aim of this study is to assess the diagnostic utility of symptoms for the yield (CRC) of colonoscopy and to compare this with the diagnostic utility of FIT when offered to symptomatic patients. Methods: We performed a systematic review search for CRC as an outcome of colonoscopy in referred symptomatic patients and separately for CRC as an outcome in symptomatic patients with a positive FIT. Results: We included 35 studies, with almost 5 million symptomatic patients. In addition, we included nine prospective studies with a positive FIT in symptomatic patients, with more than 5000 patients. In a random effect model, the pooled sensitivity of colonoscopy in symptomatic patients was very low (25%). However, the pooled sensitivity in symptomatic patients with a positive FIT was 83% and the pooled specificity 77%. A total of 75 symptomatic patients (1.4%) had a false-negative FIT. Conclusion: Adding FIT in symptomatic patients seems useful for predicting CRC as an outcome of colonoscopy. FIT seems a potential tool for an improved triage of colonoscopy in symptomatic patients.

**Abstract:**

If Colorectal cancer (CRC) is detected and treated early, the survival rate is high. This is one of the reasons that population-based screening programs for the early detection of CRC using the faecal immunochemical test (FIT) started worldwide. These programs compete with regular colonoscopy programs and increase the waiting time for symptomatic patients. However, the literature has shown that the correlation between intestinal complaints and the gain of colonoscopy is poor. The aim of this study is to assess the diagnostic utility of symptoms for the yield (CRC) of colonoscopy and to compare this with the diagnostic utility of FIT when offered to symptomatic patients. Methods: We performed a systematic review search for CRC as an outcome of colonoscopy in referred symptomatic patients and separately for CRC as an outcome in symptomatic patients with a positive FIT. We searched systematically for clinical trials or observational studies in databases, followed by hand-searching of reference lists. We used random Meta-Disc to evaluate the diagnostic performance, using the exploration of heterogeneity with a variety of test statistics and by computing the pooled estimates. Results: We included 35 studies, with almost 5 million symptomatic patients. In addition, we included nine prospective studies with a positive FIT in symptomatic patients, with more than 5000 patients. Significant heterogeneity was found for every symptom and the outcome of colonoscopy in the effect size of sensitivity, specificity, positive likelihood ratio, negative likelihood ratio and diagnostic odds ratio. In a random effect model, the pooled sensitivity of colonoscopy in symptomatic patients was very low (25%). However, the pooled sensitivity in symptomatic patients with a positive FIT was 83% and the pooled specificity 77%. A total of 75 symptomatic patients (1.4%) had a false-negative FIT. Conclusion: Adding FIT in symptomatic patients seems useful for predicting CRC as an outcome of colonoscopy. FIT seems a potential tool for an improved triage of colonoscopy in symptomatic patients.

## 1. Introduction

Colorectal cancer (CRC) is the second most common cancer in the world [1,2]. Early detection and subsequent treatment of CRC increases the chance of survival. The 5-year survival rate in stage I is more than 90% and in stage IV just 10% [3,4]. Presumed predictive symptoms for CRC such as rectal bleeding, change in bowel habit and abdominal pain are non-specific and common in the general population [5,6,7,8,9]. The majority of these symptomatic patients (60–80%) do not have CRC. This makes it challenging to differentiate who to refer for colonoscopy. Subsequently, many patients are unnecessarily exposed to an unpleasant and invasive procedure with risk of complications [10,11,12]. Besides these risks, there are unnecessarily high costs involved [13].

The use of the guaiac faecal occult blood test (gFOBT) can reduce CRC mortality in asymptomatic population screening [14]. Additionally, major disadvantages have been identified (e.g., the test is not sensitive to small bleeds, specificity can be affected by diet or drugs, participant acceptance can be low, means of laboratory quality control are limited, and there is a fixed hemoglobin concentration cutoff determining positivity) [15]. For all these reasons, gFOBT seems obsolete to use for screening for CRC currently [16]. The current European guidelines recommended the new faecal immunochemical test (FIT), also called immunochemical fecal occult blood test (iFOBT), for CRC screening purposes [17]. Many studies have shown that FIT is superior to gFOBT for population-based CRC screening. The sensitivity for detecting CRC and advanced adenoma is higher; the participation rate is also higher. Another advantage of iFOBT is that the cut-off level of the hemoglobin concentration that defines a positive test can be defined [18]. The results of population screening with FIT in the Netherlands have shown a sensitivity of 65% and a specificity of 92% [19]. These programs compete with regular colonoscopy capacity and increase the waiting time for symptomatic patients.

If the expected yield of colonoscopy based on patient symptoms is poor and the sensitivity of FIT is high, the question arises whether FIT should be part of the diagnostic workup in symptomatic patients, to stratify patients on the waiting list based on expected yield of the colonoscopy.

The NICE guidelines recommend FIT for adoption in primary care to guide referral in people presenting with certain clinical signs and symptoms that may suggest colorectal cancer, but do not meet the following criteria:

(1) aged 40 and over with unexplained weight loss and abdominal pain;
(2) or aged 50 and over with unexplained rectal bleeding;
(3) or aged 60 and over with iron-deficiency anaemia or changes in bowel habit; or tests show occult blood in their faeces.

In addition, CRC should be considered in adults with a rectal or an abdominal mass and in adults aged under 50 with rectal bleeding and any of the following unexplained symptoms: abdominal pain, change in bowel habit, weight loss, iron-deficiency anaemia [20].

According to these criteria, most symptomatic patients need to undergo a colonoscopy within two weeks after referral. It is questionable if this is really necessary.

The aim of this systematic review is to assess and discuss the diagnostic utility of symptoms for the yield (CRC) of colonoscopy and to compare this with the diagnostic utility of FIT when added to the workup of symptomatic patients.

## 2. Materials and Methods

We performed two literature searches. In the first literature search, studies were included that aimed to assess the yield of colonoscopy in terms of CRC when the indication for colonoscopy was based on the following symptoms: rectal bleeding, change in bowel habits, obstipation, diarrhea, abdominal pain, weight loss, iron-deficiency anemia or a palpable abdominal mass. In the second literature search, studies were included that aimed to assess the yield of colonoscopy in terms of CRC when the indication for colonoscopy was based on an positive iFOBT in addition to symptoms.

The systematic review followed the recommendations of the Preferred Reporting Items for Systematic Reviews and Meta-Analyses (PRISMA). The protocol has not been registered.

### 2.1. Search Strategy

Guidelines, PubMed, Medline, Tripdatabase and Cochrane databases were searched systematically from 1985 to May 2017 for clinical trials or observational studies, followed by hand-searching of reference lists. A combination of MeSH terms and text words was used. The full search strategy is given in additional file 1. Bibliographies and references of included studies, review articles and clinical guidelines were also searched. Only studies in English or Dutch were selected.

The patients in the included studies in both searches were referred for colonoscopy to exclude CRC. Studies that investigate the diagnostic accuracy of symptoms, signs and diagnostic tests in relation to CRC were used with a colonoscopy or barium enema as the reference standard.

All studies that used FIT in symptomatic patients were included. Papers that did not differentiate between CRC, polyps and/or IBD were excluded.

### 2.2. Quality Assessment

We extracted data of all papers regarding setting and design, study population, test characteristics and test results. Methodological quality was assessed with the quality assessment of a diagnostic accuracy studies (QUADAS) tool, which is recommended by the Cochrane Diagnostic Reviewers Handbook. This modified version consists of 14 items on methodological characteristics that have the potential to introduce bias. Items were scored as positive (no bias), negative (potential bias) or unclear. The QUADAS summary is shown in Table 1.

### 2.3. Data Extraction

The true positives, true negatives, false negatives and false positives of each individual symptom were extracted from the included articles. If these data were not mentioned, we tried to retrieve and calculate this with the necessary numbers. The study was excluded if we could not compute the data. Data extraction was conducted by one reviewer (BvD) and checked by a second reviewer (HJB).

### 2.4. Statistical Analysis

We used Meta-Disc to evaluate the diagnostic performance. The degree of heterogeneity of sensitivity specificity positive and negative likelihood and the diagnostic odds ratio among the studies was investigated using the likelihood ratio Chi-square test and the Q statistic. When the Q test was statistically significant, we changed from a fixed effect model to a random effects model. The I2 index was used for quantifying potential heterogeneity between the studies. In general, I2 = 25%, 50% and 75% corresponds with low, medium, and high heterogeneity, respectively. Stratified analyses were performed to investigate factors that could contribute to diagnostic performance across studies including prospective studies only, studies with only colonoscopy as reference standard, and studies including patient >30 years old.

## 3. Result

### 3.1. Publication Searching Results

**Figure 1:**
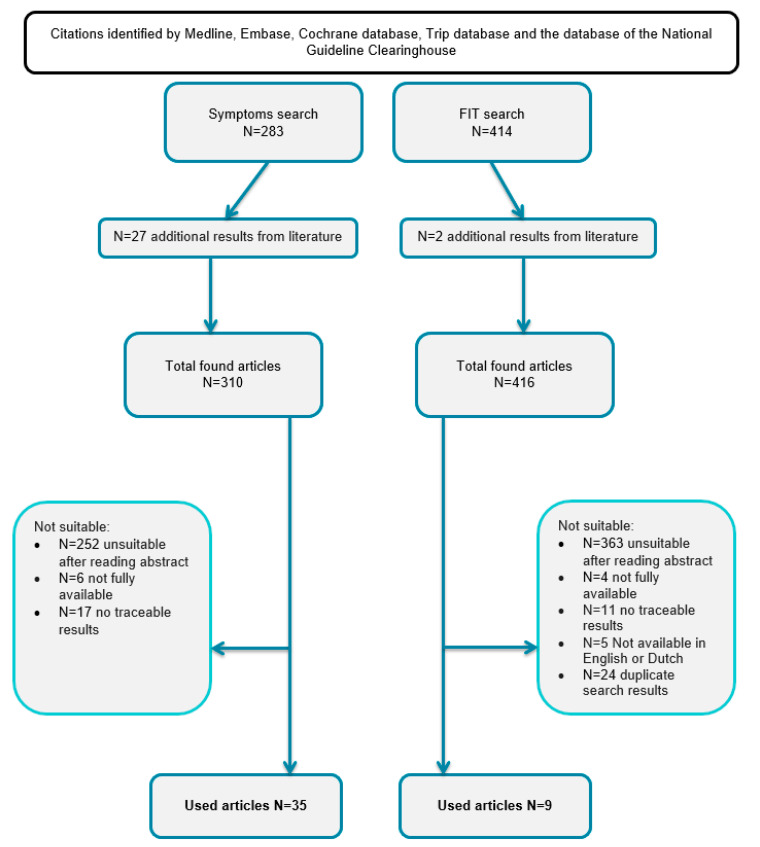
The publication searching procedure.

We included 35 studies 26 prospective and 9 retrospective studies with a total of 4,833,056 symptomatic patients [21,22,23,24,25,26,27,28,29,30,31,32,33,34,35,36,37,38,39,40,41,42,43,44,45,46,47,48,49,50,53,54,55,56,58,59]. In addition, we included nine prospective studies with a positive FIT in symptomatic patients, with a total of 5296 patients [24,34,47,51,52,53,54,55,56]. The publication searching procedure is demonstrated in Figure 1 in a consort diagram.

Further study characteristics can be found in the Appendix B.

### 3.2. Statistical Heterogeneity

For every symptom, significant heterogeneity was found in the effect size of sensitivity, specificity, positive likelihood ratio, negative likelihood ratio and diagnostic odds ratio. These effect sizes were pooled by the random effect model.

### 3.3. Symptoms

Table 2 shows the pooled sensitivity, specificity, positive and negative likelihood and diagnostic odds ratio of the studied symptoms. The pooled sensitivity for CRC in symptomatic patients was 25%. A total of 14,159 symptomatic patients had been diagnosed with CRC, which means a true positive of 0.3%.

### 3.4. Symptomatic Patients and FIT

Table 3 shows the pooled sensitivity, specificity, positive and negative likelihood ratio for the FIT for patients with symptoms. Table 4 shows the Statistical heterogeneity evaluation. A total of 75 symptomatic patients (1.4%) had a false-negative FIT. Strikingly, only 35% of all patients with rectal bleeding also had a positive FIT.

## 4. Discussion

This study aimed to assess the diagnostic utility of symptoms for the yield (CRC) of colonoscopy and compared this with the diagnostic utility of FIT when added in symptomatic patients. Our study showed that using symptoms to predict CRC as an outcome of colonoscopy in symptomatic patients seems not useful. The pooled sensitivity for CRC in symptomatic patients is very low: 25%; this ranges from 5.5% in patients with an abdominal mass to 33% in patients with abdominal pain. On the other hand, the pooled specificity of a colonoscopy is 97%, ranging from 64% with diarrhea to 99% with weight loss. In our meta-analysis, only 0.3% of the symptomatic patients had been diagnosed with CRC.

On the contrary, the yield of colonoscopy if FIT is added in symptomatic patients is high. When the FIT is added in the diagnostic workup of a symptomatic patient, there is both an acceptable sensitivity (83%) and specificity (77%). FIT seems to be a potential tool for a better triage for colonoscopy in symptomatic patients.

The study is a review and meta-analysis of previous studies; the yield of colonoscopy when the indication is based on so-called “alarm features for CRC” is low.

The strength of this study is that we put it in a clear overview and the extensive meta-analysis indicates clear numbers. Another strength and novelty of this review is that we did a meta analysis on the yield of colonoscopy when a positive FIT is added to symptoms.

The study methods followed the traditional scheme for systematic reviews. A broad selection of symptoms was chosen to ensure all relevant studies were included. We did not use many exclusion criteria, so we included a wide variety of studies. The strengths of this research also entail some implications. Because of the wide variety of studies, the total study period is very long. We chose to focus on single symptoms, because otherwise too many combinations are possible, leaving fewer studies to compare. Although it seems plausible that patients with more symptoms have a higher chance of CRC, other symptoms may actually have been present but were not reported in the included studies.

Most of the symptoms are very subjective and no clear symptom description was given. For the sake of clarity in this paper, and to include as many studies as possible per symptom, we did not distinguish between different terminologies of the same symptom. Because we included a wide variety of studies, there was considerable heterogeneity between studies. The larger studies are all from electronic databases; their results are not directly comparable to smaller studies. The heterogeneity may also be due to the unclear symptom description and differing severity and variations in referral rates. Additionally, and probably most importantly, every included study showed low sensitivity for all the symptoms. The low sensitivity is probably explained by the fact that the symptoms studied are common in the general population and may have many other causes besides a CRC (3—7). Doctors should be aware that the yield of colonoscopy in terms of CRC when the indication is only based on symptoms is low. The majority of these patients are unnecessarily exposed to an unpleasant and invasive procedure with a risk of complications and needless costs involved [13]. Furthermore, waiting lists for colonoscopy are becoming longer since the introduction of the population screening for CRC [13].

The NICE guideline was adjusted in 2017, which recommends FIT for people with unexplained symptoms that do not meet the criteria for a suspected cancer pathway referral. The recommendation after a positive FIT is to perform a colonoscopy within two weeks [20]. By these criteria, most symptomatic patients are covered by the suspected CRC pathway. Is it really necessary to consider all of these patients as suspected for CRC? 

## 5. Conclusions

We describe a true-positive rate of CRC as an outcome of colonoscopy in 0.3% of these patients. We would suggest using the FIT as a triage tool for colonoscopy, to increase the sensitivity of symptoms. Patients with symptoms and a negative FIT are probably better off with a consultation of a gastroenterologist before planning for colonoscopy, as there may well be another underlying cause. Further research is needed to test this triage system.

## Figures and Tables

**Table 1:** The QUADAS method.

	1	2	3	4	5	6	7	8	9	10	11	12	13	14
Bafandeh 2008 [21]	+	+/−	+	+	+	+	?	+	+	−	−	+	+	+
Bjerregaard 2006 [22]	+	+	+	+	+	+	+	+	+	+/−	+/−	+	?	+
Brewster 1994 [23]	+	+/−	−	+	+	−	+	−	+	+	+/−	+	−	−
Farrands 1985 [24]	+	+/−	+/−	+	+	+/−	+	+/−	+	+	+/−	+	?	−
Selvachandran 2002 [25]	+	+/−	+	+	+	+/−	+	+	+	+	+/−	+	?	+
Steine 1994 [26]	NB													
Tan 2002 [27]	+	+/−	+	+	+	+	+	+/−	+	+/−	+/−	+	?	−
Tate 1988 [28]	+	+/−	+	+	+	+/−	+	+/−	+	−	+/−	+	?	+
Thompson 2007 [29]	+	+	+/−	+	+	+	+	+	+	+	−	+	?	−
Thompson 2008 [30]	+	+	+/−	+	+	+/−	+	+/−	+	+	+/−	+	?	−
Zarchy 1991 [31]	NB													
Panzuto 2003 [32]	+	+	+	+	+	+/−	+	+	+	+	+/−	+	?	+
Curless 1994 [33]	+	+	+	+	+	−	+	+/−	+	−	+	−	?	+
Jensen 1993 [34]	+	+/−	+/−	+	+	+/−	+	+/−	+	+/−	−	+	?	?
Patel 2016 [35]	+	+/−	+	+	+	+/−	+	+/−	+	−	+/−	+	?	?
Cheong 2000 [36]	+	+/−	+	+	+	+/−	+	+/−	+	+/−	+/−	+	?	?
Hipsley-Cox 2012 [37]	+	+	+	?	+	+/−	+	+	+/−	−	?	+	−	+
Simpkins 2017 [38]	+	+	+	+	+	+/−	+	+	+	+	+	+	?	+
Hamilton 2009 [39]	+	+	+	+	+	+	+	+	+	+	+/−	+	?	?
Koning 2015 [40]	+	+	+	+	+	+/−	+	+	+	+	−	+	?	?
Hamilton 2005 [41]	+	+	?	?	?	?+	+	+/−	?	−	?	+	−	+
De Bosset 2002 [42]	+	+	+	+	+	+	+	+	+	+	−	+	?	?
Cai 2015 [43]	+/−	+/−	+	+	+	+/−	+	+/−	+	+	−	+	?	?
Pepin 2002 [44]	+	+	+	+	+	+	+	+/−	+	+/−	+/−	+	?	?
Flashman 2004 [45]	+/−	+	+	+	+	+/−	+	+	+	+	+	+	?	+
Du toit 2006 [46]	+	−	+	+	+	−	+	+/−	+	+	−	+	?	?
Nakama 2000 [47]	+	+	+	+	+	+	+	+	+	+	−	−	?	?
Wauters 2000 [48]	+	+/−	+	+	+/−	+/−	+	+/−	+/−	+/−	+/−	?	?	−
Ahmet 2005 [49]	+/−	+	+	+	+	−	+	+	+	+/−	+/−	+	−	+
Brenna 1990 [50]	+	+/−	+	+	+	−/+	+	−	+	+	−	+	−	+
Mc donald 2013 [51]	+	+	+	+	+	+	+	+	+	?	?	+	?	−
Elias 2016 [52]	+	+	+	+	+	+/−	+	+	+	+	+	+	?	+
Hogberg 2017 [53]	+	+	+	+	−	−	+	+	+	+	−	+	?	+
Mowat 2016 [54]	+	+/−	+	+	+	+	+	+	+	+	+	+	?	+
Cubiela 2014 [55]	+	+	+	+	+	+	+	+/−	+	+	+	+	?	+
Cubiela 2016 [56]	+	+	+	+	+	+	+	+	+	+	+	+	?	+
Rodriguez 2015 [57]	+	+	+	+	+	+	+	+	+	+	+	+	?	+
Law 2014 [58]	+	+/−	+	+	+	+	+	+/−	+	+	?	+	?	+

+ = no bias; − = bias −/+ = potential bias; ? = bias unclear. 1 = valid selection, representative patients, 2 = selection clearly described 3 = adequate reference test 4 = target condition did not change between tests, 5 = all/random selection received verification via reference test, 6 = all received same test, 7 = index test not part of reference 8 = index test described in detail 9 = reference test described in detail. 10 = blinded to reference standard, 11 = blinded to index test, 12 = clinical data available as normal, 13 = no missing/uninterpretable data 14 = withdrawals explained.

**Table 2:** The pooled sensitivity, specificity, positive and negative likelihood and diagnostic odds ratio of the studied symptoms.

	Pooled Sensitivity	Pooled Specificity	Pooled Likelihood +	Pooled Likelihood −	Pooled DOR
Changed bowel habit	0.235 (0.226–0.244)	0.974 (0.974–0.973)	1.603 (1.194–2.151)	0.841 (0.773–0.914)	1.979 (1.158–3.382)
Diarrhea	0.192 (0.182–0.202)	0.635 (0.625–0.644)	0.747 (0.278–2.008)	1.119 (0.684–1.834)	0.650 (0.139–3.050)
Obstipation	0.266 (0.254–0.277)	0.888 (0.885–0.891)	1.168 (0.754–1.809)	1.022 (0.900–1.161)	1.177 (0.698–1.986)
Anemia	0.285 (0.274–0.297)	0.985 (0.985–0.984)	2.661 (1.911–3.704)	0.818 (0.707–0.947)	3.490 (2.523–4.826)
Abdominal pain	0.329 (0.319–0.340)	0.741 (0.743–0.738)	1.176 (0.825- 1.676)	1.006 (0.894–1.133)	1.161 (0.703–1.918)
Weight loss	0.116 (0.110–0.123)	0.986 (0.987–0.986)	2.358 (1.684–3.300)	0.902 (0.863–0.943)	2.741 (1.835–4.094)
Rectal blood loss	0.313 (0.305–0.322)	0.963 (0.963–0.963)	2.037 (1.286–3.227)	0.837 (0.767–0.913)	2.501 (1.337–4.677)
Abdominal mass	0.055 (0.030–0.090)	0.969 (0.963–0.974)	1.780 (0.798–3.969)	0.991 (0.950–1.033)	1.018 (0.364–2.843)
Cumulative symptoms	0.248 (0.244–0.251)	0.972 (0.971–0.972)	1.620 (1.356–1.936)	0.923 (0.896–0.951)	1.792 (1.389–2.311)

**Table 3:** The pooled sensitivity, specificity, positive and negative likelihood ratio for the FIT for patients with symptoms.

	Pooled Sensitivity	Pooled Specificity	Pooled Likelihood +	Pooled Likelihood −	Pooled DOR
FIT	0.830 (0.792–0.863)	0.765 (0.755–0.775)	3.886 (2.640–5.721)	0.155 (0.086–0.278)	27,025 (18,509–39,459)

**Table 4:** Statistical heterogeneity evaluation FIT.

Effect Size	Chi Square	I2%	*p*
Sensitivity	63.19	82.6	0.000
Specificity	725.01	98.5	0.000
Positive likelihood ratio	589.80	98.1	0.000
Negative likelihood ratio	39.82	72.4	0.000
Diagnostic odds ratio	12.80	14	0.307

## Data Availability

There are no data created.

## Appendix A

((((“Colonic Neoplasms”[tiab] OR “Colonic Neoplasm”[tiab] OR “Colon Neoplasms”[tiab] OR “Colon Neoplasm”[tiab] OR “Colon Cancers”[tiab] OR “Colon Cancer”[tiab] OR “Colonic Cancers”[tiab] OR “Colonic Cancer”[tiab] OR “Cancer of Colon”[tiab] OR “Cancer of the Colon”[tiab] OR “Colon carcinoma”[tiab] OR “Colonic malignancy” OR “colonic tumor”[tiab] OR “colonic tumors”[tiab] OR “colon tumor”[tiab] OR “colon tumors”[tiab])) OR (“Colonic Neoplasms”[Mesh])) OR (((“Rectal Neoplasms”[Mesh])) OR (“Rectal neoplasms”[tiab] OR “Rectal neoplasm”[tiab] OR “Rectal cancer”[tiab] OR “Rectal cancers”[tiab] OR “Rectal carcinoma”[tiab] OR “Rectal malignancy” OR “Rectal tumor”[tiab] OR “Rectal tumors”[tiab] OR “Cancer of the Rectum”[tiab] OR “Rectum cancer”[tiab])) OR (((“Colorectal neoplasms”[tiab] OR “Colorectal neoplasm”[tiab] OR “Colorectal cancer”[tiab] OR “Colorectal cancers”[tiab] OR “Colorectal carcinoma”[tiab] OR “Colorectal malignancy” OR “colorectal tumor”[tiab] OR “colorectal tumors”[tiab])) OR (“Colorectal Neoplasms”[Mesh]))) AND (referral OR referrals) AND (primary care).

(“rectal bleeding” OR “gastrointestinal hemorrhage” OR hematochezia “Gastrointestinal Hemorrhage”[Mesh]) OR “change of bowel habit” OR “abdominal pain” OR (“abdominal mass” OR mass) OR (“weight loss” OR “weight reduction” OR “Weight Loss”[Mesh]) OR (obstipation OR constipation) OR (anemia OR “iron deficiency anemia” OR “Anemia”[Mesh]) OR “Diarrhea”[Mesh] OR symptomatic AND (“Colonic Neoplasms”[tiab] OR “Colonic Neoplasm”[tiab] OR “Colon Neoplasms”[tiab] OR “Colon Neoplasm”[tiab] OR “Colon Cancers”[tiab] OR “Colon Cancer”[tiab] OR “Colonic Cancers”[tiab] OR “Colonic Cancer”[tiab] OR “Cancer of Colon”[tiab] OR “Cancer of the Colon”[tiab] OR “Colon carcinoma”[tiab] OR “Colonic malignancy” OR “colonic tumor”[tiab] OR “colonic tumors”[tiab] OR “colon tumor”[tiab] OR “colon tumors”[tiab] OR “Colonic Neoplasms”[Mesh] OR “Rectal Neoplasms”[Mesh] OR “Rectal neoplasms”[tiab] OR “Rectal neoplasm”[tiab] OR “Rectal cancer”[tiab] OR “Rectal cancers”[tiab] OR “Rectal carcinoma”[tiab] OR “Rectal malignancy” OR “Rectal tumor”[tiab] OR “Rectal tumors”[tiab] OR “Cancer of the Rectum”[tiab] OR “Rectum cancer”[tiab] OR “Colorectal neoplasms”[tiab] OR “Colorectal neoplasm”[tiab] OR “Colorectal cancer”[tiab] OR “Colorectal cancers”[tiab] OR “Colorectal carcinoma”[tiab] OR “Colorectal malignancy” OR “colorectal tumor”[tiab] OR “colorectal tumors”[tiab] OR “Colorectal Neoplasms”[Mesh]) AND (FIT[tiab] OR iFOBT[tiab] OR “Fecal immunochemical Test”[tiab]).

## Appendix B

**Study**	**Type**	**N**	**Location**	**Study** **Period**	**Characteristics of Method**
Bafandeh 2008 [21]	Prospective	480	Imam Hospital, Tabriz University of medical sciences, Iran.	2 years	- long lasting lower gastrointestinal tract symptoms - every age
Bjerregaard 2006 [22]	Cross-sectional, prospective	2172	Surgical outpatient clinics of two public Danish hospitals: Randers Central Hospital (RCH) and Aarhus University Hospital (AUH) in Aarhus County, Denmark.	16 months	- >40 years old - referred by general practitioners - symptoms consistent with CRC - Colonoscopy - Questionnaire about symptoms
Brewster 1994 [23]	Prospective	462	Leith Hospital, Edinburgh, UK	3 years	- Referred for barium enema → also flexible sigmoidscopy.
Farrands 1985 [24]	Prospective	152	Southampton General hospital, UK	-	- Gastro-intestinal symptoms suggestive of colorectal disease. - 101: Rectal examination, proctoscopy and sigmoidscopy → FIT → colonoscopy or bariumeneme - 51: FIT → bariumeneme or colonoscopy
Selvachandran 2002 [25]	Prospective	2268	Leighton hospital, Crewe, UK	2 years	- Distal colonic symptoms - Referred by GP for endoscopic assessment - Questionnaire
Tan 2002 [27]	Prospective Cross-sectional	485	University Hospital, Kuala Lumpur, Malaysia.	22 months	- Referred by GP for colonoscopy - Questionnaire
Tate 1988 [28]	Prospective	137	Royal South Hampshire Hospital, Southampton, UK 52 GP’s.	1 year	- Referral by GP suspected for colonic neoplasia
Thompson 2007 [29]	Prospective, observational	8529	Portsmouth, single surgical outpatient clinic, UK	12 years	- All consecutive patients with lower gastrointestinal symptoms - Everyone sigmoidscopy - When doctor decided: colonoscopy/Barium enema?
Thompson 2008 [30]	Prospective, observational	16,433	St Mary’s Hospital and two peripheral hospitals in and near Portsmouth, UK	16 years	- newly referred patients with symptoms or signs of colorectal cancer. - Sigmoidscopy either alone or followed by bariumenema, colonoscopy or CT colonography.
Panzuto 2003 [32]	Prospective	280	Lazio, Italy; 159 GP’s.	8 weeks	- consecutive outpatients with symptoms considered suspicious for the presence of a colon disease to rule out the presence of CRC. - Colonoscopy or barium enema - Trained GP’s - Exclusion: previous diagnoses of colorectal disorders or a recent large bowel examination
Curless 1994 [33]	Retrospective	123 +125 control <70 year 150 + 148 control <70 year	Hospitals of Gateshead and Newcastle Health Districts, UK	1 year	- histological diagnosis of colorectal adenocarcinoma - Within 2 weeks interview - Exclusions: previous diagnosis of colorectal adenoma or carcinoma, known colitis, non-whites, and those dying before interview - Controls: matched with sex and age
Jensen 1993 [34]	Prospective	194	Varberg Hospital, Sweden	-	- symptoms indicating colorectal disease, referred by GP for double-contrast barium enema (DCE) - fecal occult blood test and rectosigmoidoscopy before the DCE
Patel 2016 [35]	Retrospective	197	West Suffolk Hospital, Suffolk, UK	6 years	- primary care referrals for suspected colorectal malignancy - <50 years
Cheong 2000 [36]	Prospective	375	Hospital University Kebangsaan Malaysia, Kuala Lumpur	1 year	- All patients undergoing colonoscopy
Hippisley-Cox 2012 [37]	Cohort study using data from 375 UK QResearch^®^ general practices for development and 189 for validation.	4.1 million person years	All practices in England and Wales that had been using their EMIS (Egton Medical Information System) computer system for at least a year.	10 years	- 30–84 years - free at baseline from a diagnosis of colorectal cancer and without rectal bleeding, abdominal pain, appetite loss, or weight loss in the previous 12 months. - colorectal cancer recorded in the next 2 years
Simpkins 2017 [38]	Prospective	1981	The McMaster University Medical Center, and St. Joseph’s Healthcare, Hamilton, Canada	4 years,	- Lower GI symptoms. - Assessors were blinded to symptom status. - Reference: histopathological confirmation - Controls: patients without CRC.
Hamilton 2005 [41]	Population based Case-control, retrospective	2093	21 GP’s, Exeter, Devon, UK	4 years	- full medical record for 2 years before diagnosis was coded using the International Classification of Primary Care-2.
Koning 2015 [40]	Cross-sectional	3855	Julius General Practitioners’ Network (JGPN) database Utrecht area, Netherlands	5 years	- Referred for colonoscopy by GP - Data were obtained from the Julius General Practitioners’ Network (JGPN) database. - Exclusion: history of CRC
Hamilton 2008 [59]	Case-control	51,508	Database UK	6 years	- data from The Health Improvement Network (electronic medical records from GP practices) - >30 years with CRC
Hamilton 2009 [39]	Case-control	43,791	Database UK	5 years	- Data were provided by The Health Improvement Network - 2 years of data - Patients >30 years with CRC
De Bosset 2002 [42]	Prospective, observational	1188	Two district hospitals; Porrentruy and Dele mont, one university hospital and its outpatient department in Zurich and two gastroenterology practices in Delemont and Yverdon in Switzerland.	17 months	- Consecutive patients referred for diagnostic Colonoscopy Swiss criteria developed by the Rand Corporation/University of California at Los Angeles (RAND/UCLA) panel method
Cai 2002 [43]	Retrospective	580	First Affiliated Hospital of Chongqing Medical University, southwest China.	6 years	- Colonoscopic findings from patients with chronic abdominal pain, chronic diarrhea and constipation systematically analyzed in retrospect. - 13–77 years old
Pepin 2002 [44]	Retrospective	563	Moffitt- Long Hospital (MLH), General Hospital (SFGH) and the San Francisco Veterans Administration Medical Center (SFVAMC), San Francisco, US	3 years	- endoscopic database was searched systematically to identify all patients who underwent sigmoidoscopy (SIG) or colonoscopy (COL) for constipation.
Flashman 2004 [45]	Prospective	249	Queen Alexandra Hospital, Cosham, Portsmouth	1 year	- All patients with bowel cancer; all patients referred on the basis of the two week standard and to a routine colorectal surgical outpatient clinic.
Du toit 2006 [46]	Cohort	265	GP practice in the UK	10 years	- Participants: Patients aged 45 or more with new onset rectal bleeding, irrespective of other symptoms. - Main outcome: Percentage of participants in whom colorectal cancer or colonic adenoma was identified after investigation of the bowel.
Nakama 2000 [47]	Cross-sectional	9625	Japan	4 years	- Medical check up - Colonoscopy + Fobt (for screening) - 2 groups: rectal bleeding or not - No other symptoms
Wauters 2000 [48]	Retrospective	83,890	A network of sentinel practices, Belgium, (covering 1% of the population),	1 year	- Patients presenting with rectal bleeding - Reference standard: CRC
Ahmed 2005 [49]	Prospective	563	Scotland	1 year	- consecutive individuals with a positive FOB test in the Scottish arm of the national colorectal cancer screening pilot - standard questionnaire to elicit gastrointestinal symptoms;
Brenna 1990 [50]	Prospective	833	Trondheim Regional and University Hospital, Trondheim, Norway	1 year	- Referred for colonoscopy
Mc donald 2013 [51]	Prospective	280	Ninewells Hospital and Medical School, Dundee, UK	2 years	- referred from primary care for endoscopic examination of the lower gastrointestinal tract - Symptomatic patients - single sample faecal collection for Hb concentration measurement. - >16 year
Elias 2016 [52]	Prospective	810	266 Dutch primary care practices	3 years	- SCD-suspected patients referred for endoscopy to develop a diagnostic model for SCD with routine clinical information, with faecal calprotectin POC (quantitatively in μg/g faeces) and/or POC FIT results (qualitatively with a 6 μg/g faeces detection limit). - SCD: colorectal cancer (CRC), inflammatory bowel disease, diverticulitis, or advanced adenoma (>1 cm).
Hogberg 2017 [53]	Prospective	373	Four health care centres in Region Jamtland Harjedalen, Sweden	1 year	- consecutive patients that received a FIT or a FC test ordered by a primary care physician. - samples for FITs, FC tests, full blood counts and iron-deficiency tests. - Physicians were instructed to refer patients with a positive FIT or FC test (cut-off 100 lg/g) for bowel imaging. - The patients’ presenting symptoms were recorded.
Mowat 2016 [54]	Prospective	1031	Ninewells Hospital and Medical School, Dundee, UK	5 months	- All adult patients referred for investigation of bowel symptoms - GPs: FHb and FC, full blood count, urea and electrolytes and CRP and record the presenting symptoms via the NHS Tayside electronic test requesting software. - More than one presenting symptom → attributed to one. - FHB detectable and >10.
Cubiela 2014 [55]	multicentre, prospective, blind study	787	two tertiary hospitals in northern Spain.	7 months	- patients referred for a diagnostic colonoscopy, patients with NICE and SIGN referral criteria. - All patients one FIT determination (OCsensor™) - (CRISP) questionnaire was used to record symptoms - Exclusion: age under 18, pregnancy, asymptomatic individuals for CRC screening, patients with a history of colonic for surveillance colonoscopy, patients requiring hospital admission, patients whose symptoms had ceased within 3 months before evaluation
Cubiela 2016 [56]	Prospective cross-sectional study	3053	Complexo Hospitalario Universitario de Ourense, Spain.	19 months	- consecutive patients with gastrointestinal symptoms referred for colonoscopy In the derivation cohort, assessed symptoms, NICE referral criteria, levels of faecal haemoglobin and calprotectin, blood haemoglobin, and serum carcinoembryonic antigen before colonoscopy. - Exclusion: age under 18, pregnancy, asymptomatic individuals for CRC screening, patients with a history of colonic for surveillance colonoscopy, patients requiring hospital admission, patients whose symptoms had ceased within 3 months before evaluation
Steine 1994 [26]	Prospective	1852	Oslo, Norway	-	- referred from primary health care for a double-contrast barium enema
Zarchy 1991 [31]	Prospective	794	large multispecialty medical group, LA	-	- Physicians completed a form before ordering a double-contrast barium enema, listing information about patient history, symptoms, and objective findings, including the results of a complete blood count, stool hemoccult, and sigmoidoscopy
Rodriguez 2015 [57]	Prospective	1054	Bellvitge UniversityHospital Spain	25 months	- symptomatic patients referred for a colonoscopy who provided a sample for faecal immunochemical testing - >18 years - Exclusion: adenoma surveillance andpostoperative surveillance of CRC. Hospitalized patients and those with a history of previous colectomy, IBD and polyp syndrome, incomplete colonoscopies were included only if the cause was a stenosing neoplasm.
Law 2014 [58]	Cross-sectional, prospective	1013	University Malaya, Kuala Lumpur, Malaysia	20 months	- symptomatic adults referred for an index colonoscopy. - Questionnaire - Complete examination
